# Correction to: Phytochemical library screening reveals betulinic acid as a novel Skp2‐SCF E3 ligase inhibitor in non–small cell lung cancer

**DOI:** 10.1111/cas.16163

**Published:** 2024-03-20

He D‐H, Chen Y‐F, Zhou Y‐L, et al. Phytochemical library screening reveals betulinic acid as a novel Skp2‐SCF E3 ligase inhibitor in non–small cell lung cancer. *Cancer Sci*. 2021;112: 3218–3232. https://doi.org/10.1111/cas.15005

In Figure 2, there were errors in the images (the boundaries of the bands are not smooth). The correct images (unmodified original blots) are shown below:

Figure 2C. Skp1 blot.

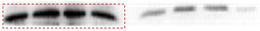

Figure 2C. Skp2 blot.

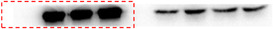

Figure 2D. Skp1 blot.

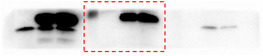

In Figure 3, there were errors in the images (the boundaries of the bands are not smooth). The correct images (unmodified original blots) are shown below:

Figure 3C. Flag blot.

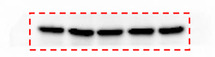

Figure 3C. HA blot.

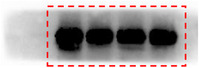

In Figure 5A, the middle panel was a duplicate of the left panel, and “LLC” and “H1299” were labeled wrongly. The correct images are shown below.

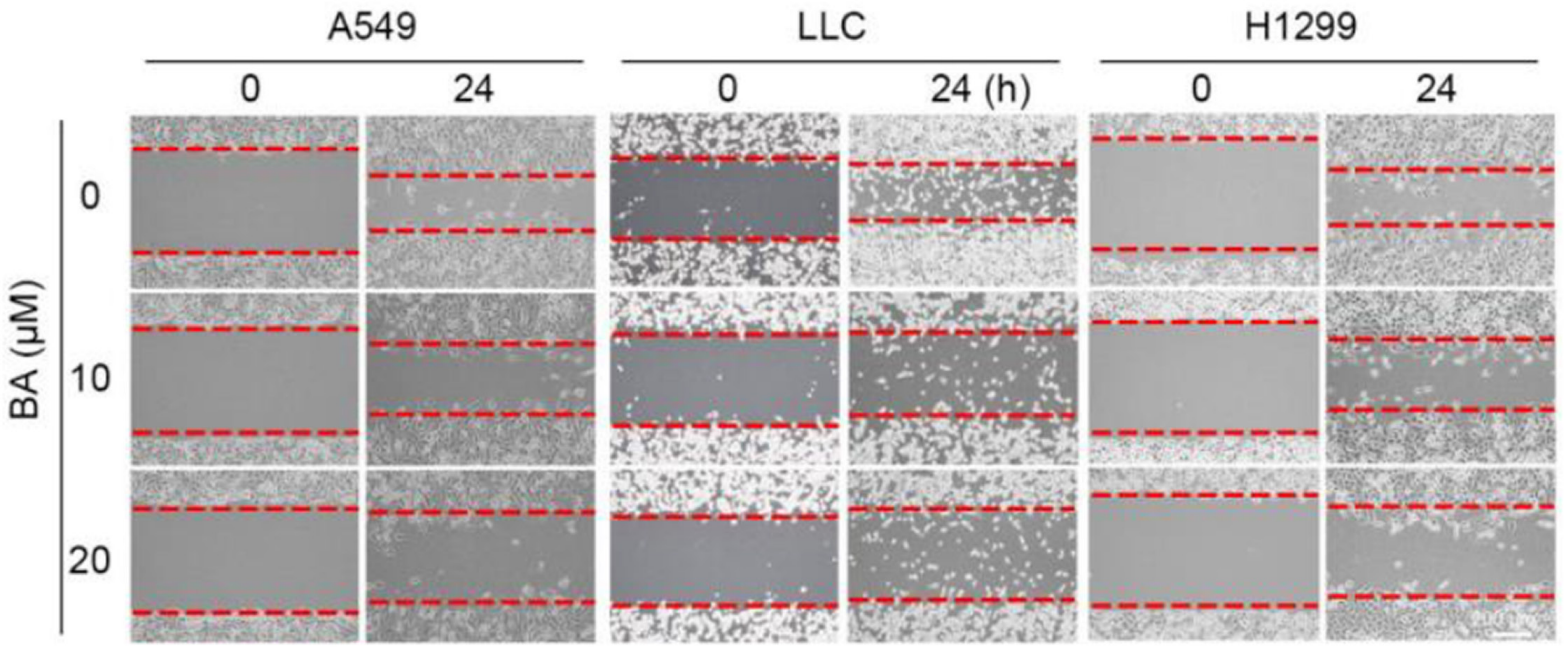

In Figure 6C, the images in the H1299‐Skp2‐24 h panel were wrong. The correct images are shown below.

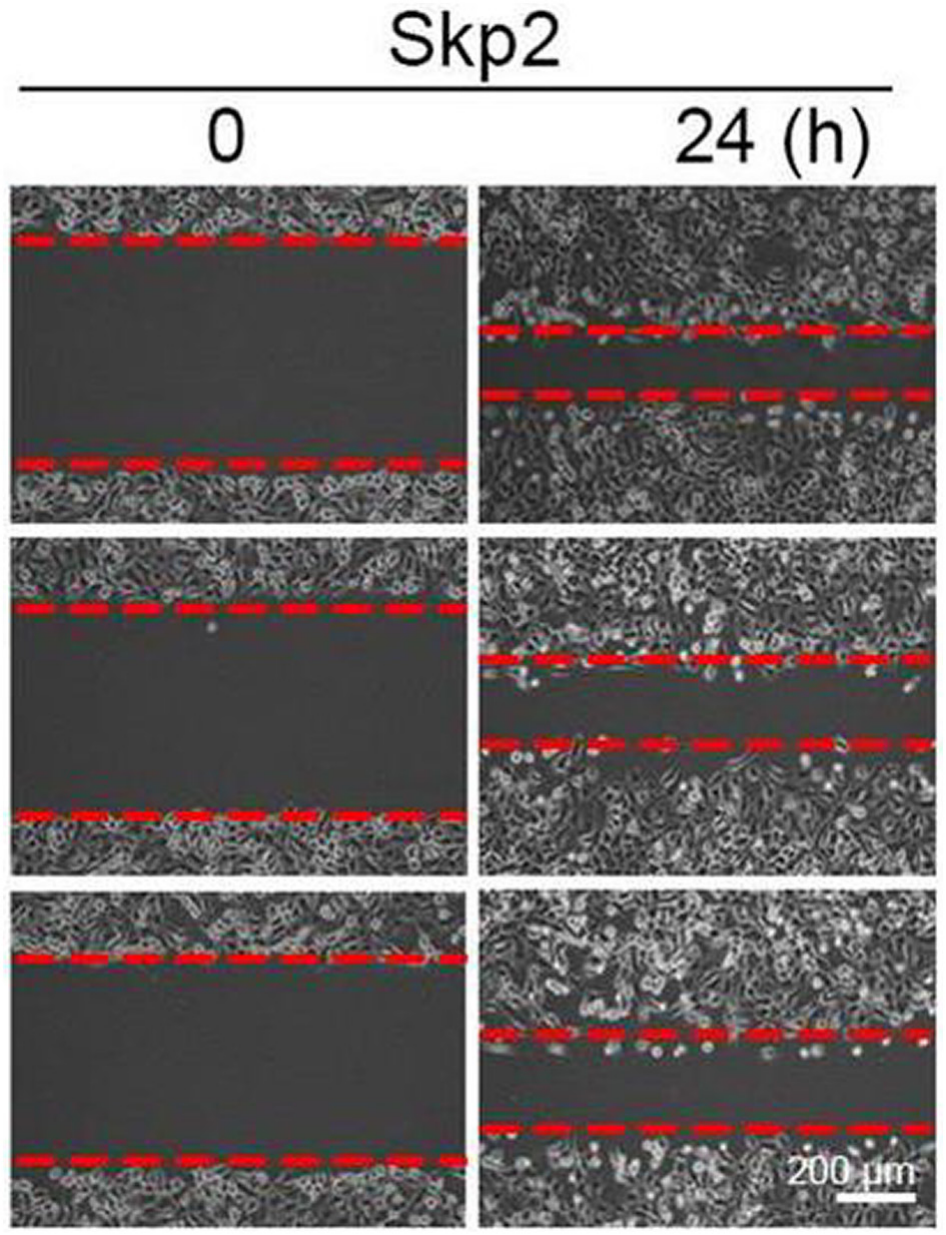

In Figure 6E, the image of BA (μM)‐0 in the A549‐Skp2 panel was wrong. The correct images are shown below.

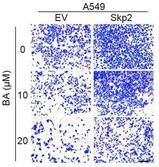

In Figure 7E, there was an error in the image (the boundaries of the bands are not smooth). The correct image (unmodified original blots) is shown below:

Figure 7E. GAPDH blot.

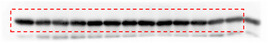

In Figure 7L, the H&E staining images in the BA‐50 group were wrong. The correct images are shown below.

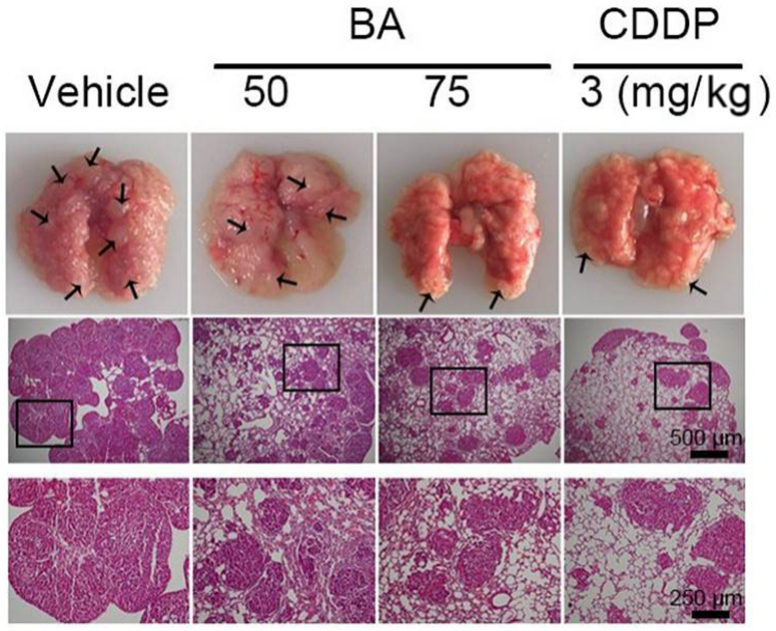

The authors apologize for the errors.

